# Commentary on: The potency of lncRNA MALAT1/miR-155 in altering asthmatic Th1/Th2 balance by modulation of CTLA4

**DOI:** 10.1042/BSR20190768

**Published:** 2020-05-04

**Authors:** Robert Foronjy

**Affiliations:** Division of Pulmonary and Critical Care Medicine, SUNY Downstate Medical Center, 450 Clarkson Avenue, MSC 19, Brooklyn, NY 11203, U.S.A.

**Keywords:** asthma, gene expression and regulation, large intervening non-coding RNA

## Abstract

Asthma is a common, allergic respiratory disorder affecting over 350 million people worldwide. One of the key features of asthma is skewing of CD4^+^ cells toward Th2 responses. This promotes the production of cytokines like IL-4 that induce IgE production resulting in the hypersecretion of mucus and airway smooth muscle contraction. Understanding the factors that favor Th2 expansion in asthma would provide important insights into the underlying pathogenesis of this disorder. Asthma research has focused on signaling pathways that control the transcription of key asthma-related genes. However, increasing evidence shows that post-transcriptional factors also determine CD4^+^ cell fate and the enhancement of allergic airway responses. A recent paper published by Liang et al. (*Bioscience Reports* (2020) **40**, https://doi.org/10.1042/BSR20190397) highlights a novel role for the long non-coding RNA metastasis-associated lung adenocarcinoma transcript 1 (MALAT1) in Th2 development in asthma. MALAT1 modulates several biological processes including alternative splicing, epigenetic modification and gene expression. It is one of the most highly expressed lncRNAs in normal tissues and MALAT1 levels correlate with poor clinical outcomes in cancer. The mechanisms of action of MALAT1 in tumor progression and metastasis remain unclear and even less is known about its effects in inflammatory disease states like asthma. The work of Liang et al. demonstrates heightened MALAT1 expression in asthma and further shows that this lncRNA targets miR-155 to promote Th2 differentiation in this disease. This insight sets the stage for future studies to examine how MALAT1 manipulation could deter allergic immune responses in asthmatic airways.

Transcriptomic analysis identified that approximately 80% of our genome is transcribed [1] yet only approximately 1–2% of these transcripts are translated into protein [2]. This indicates that a large pool of non-coding RNA transcripts are present in cells to potentially exercise exquisite control over gene expression. Long non-coding RNAs are transcripts longer than 200 nucleotides that are typically not translated into protein but instead alter cell biology by modifying chromatin structure changes, intracellular trafficking, transcription and post-transcriptional processing [3]. Researchers showed that lnc RNA could alter gene expression by modifying the effects of other non-coding RNA, such as miRNA [4]. Studies suggest that lncRNA modulates miRNA function by acting as a sponge to absorb the miRNA and prevent it from degrading its mRNA target [5,6]. Alternatively, lncRNA may antagonize the actions of miRNA by competing with them for binding to their mRNA targets [7]. As an example, the lncRNA BACE1AS stabilized the β-site amyloid precursor protein cleaving enzyme 1 (BACE1) mRNA by interfering with a specific miR-485-5p site [7]. In addition, some lncRNA are actually processed into miRNA to alter cell function. Indeed, lncRNA H19 regulates insulin signaling by generating miR-675, which targets the insulin growth factor receptor (*Igf1r*) mRNA [8]. Given their abundance and their ability to influence miRNA function, lncRNA could exert key effects on gene expression and intracellular signaling by controlling mRNA stability and protein translation.

Researchers identified abundant lncRNA expression in T-cell lineages suggesting that these transcripts play an important role in T-cell development and differentiation [9]. This could have important implications for allergic asthma where a Th2 predominant response generates cytokines, like IL-4, IL-5 and IL-13, which trigger IgE production and eosinophilic infiltration into the lung [10]. This allergic inflammation stimulates airway mucus production and smooth muscle contraction to provoke symptoms of breathlessness and respiratory distress in patients [11]. The ability of lncRNA to modulate CD4 differentiation and gene expression could impact significantly on the production of these damaging Th2 cytokines in this disease. Consistent with this theory, distinct lncRNA expression profiles were identified in T cells from subjects with difficult-to-control asthma [12]. Moreover, lncRNA levels correlate with expression of mRNA in these T cells suggesting that they exert a pivotal role in altering the effector function of these cells [13]. Thus, identifying how lncRNA change gene expression to promote Th2 skewing could provide new mechanistic insights and therapeutic targets for this disease.

In their work, Liang and Tang [14] propose that the lncRNA metastasis-associated lung adenocarcinoma transcript 1 (MALAT1) is a key determinant that promotes the Th2 phenotype in allergic asthma. Using a large and well-defined cohort of healthy and asthmatic subjects, they show up-regulation of MALAT1 expression in the blood coinciding with the down-regulation of its key target, miR-155 (Figure 1). Importantly, MALAT1 expression correlated negatively with lung function and the Th1/Th2 ratio indicating that its effects were compromising lung function by skewing toward Th2 responses. Researchers showed that the transcription factors T-bet and GATA-3 determine T-cell fate with T-bet inducing genes for Th1 development while GATA-3 turns on genes responsible for Th2 responses [15]. Competitive interactions between these two transcription factors determine gene expression and CD4^+^ differentiation. T-bet interferes with the binding of GATA-3 to its Th2 DNA targets, and GATA-3 significantly interferes with the binding of T-bet to its Th1 promoter regions [16]. Liang et al. showed that miR-155 increased T-bet and Th1 cytokines in CD4^+^ cells but had no effect on MALAT1 expression. This is important since miRNA frequently target lncRNA for degradation [17]. On the other hand, MALAT1 decreased miR-155 levels and this was accompanied by increased GATA-3 and Th2 cytokines in these CD4^+^ cells. Thus, this work establishes MALAT1 as a key orchestrator of Th2 skewing in allergic asthma.

**Figure 1:**
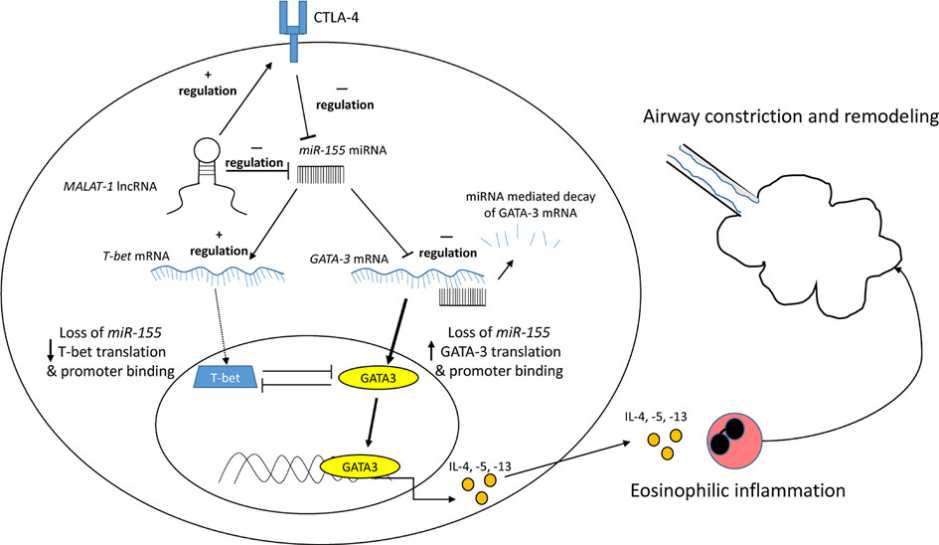
Effects of MALAT-1 on CD4+ Cells The lncRNA *MALAT-1* directly down-regulates *miR-155* miRNA. *MALAT1* also up-regulates CTLA-4 which counters the effects of *miR-155*. Under normal conditions, *miR-155* enhances *T-bet* mRNA and translation while targeting *GATA-3* mRNA for degradation. Thus, the loss of *miR-155* mediated by *MALAT1* decreases T-bet protein levels and increases GATA-3 protein levels. As a result, T-bet cannot antagonize GATA-3 promoter binding in the nucleus and GATA-3 is free to up-regulate Th2 cytokines (IL-4, -5, -13) that prime eosinophils and subsequently promote airway hyperreactivity and remodeling in asthma. Abbreviation: CTLA-4, cytotoxic T-lymphocyte antigen 4.

A particularly novel aspect of the Liang et al. [14] study was the role of cytotoxic T-lymphocyte antigen 4 (CTLA-4). This receptor protein typically negatively regulates GATA-3 levels and shifts the cell away from a Th2 phenotype [18]. However, Liang et al. [14] showed that MALAT1 increased CTLA4 expression and CTLA4 countered the Th1 differentiation induced by an miR-155 mimic or MALAT1 siRNA treatment. These findings suggest an intricate role for CTLA4 in T-cell differentiation. Consistent with the findings of Liang et al., a recent study found that CTLA-4 blockade enhanced CD8 T-cell function while limiting Th2 polarization in CD4 cells [19].What these results demonstrate is that cofactors like MALAT1 heavily influence the complex effects of CTLA-4 on T-cell differentiation. It is important to note that MALAT1’s regulation of T-bet and GATA-3 could potentially influence the development of other lymphoid cells, such as innate lymphoid cells. T-bet plays an important role in type 3 innate lymphoid cells (ILC3) [20], which counter infection and maintain epithelial integrity via their expression of IL-17 and IL-22 [21,22]. It is also required for the induction of ILC1, which releases the Th1 cytokines IFN-γ and TNF [23,24]. Conversely, GATA-3 promotes ILC2 development which produces the Th2 cytokines IL-5, IL-6 and IL-13 [25]. Innate lymphoid cells confer protection against infection [26] and promote tissue repair [27] but their aberrant activation induces asthma [28], autoimmune injury [29] and cancer [30]. Future studies will address whether MALAT1 alters innate lymphoid cell differentiation to modulate the development of these biological processes *in vivo*.

MALAT1 is conserved among species [31] and is highly expressed in normal tissues [32]. Despite its abundance, its precise biological function is not fully understood. It is thought to regulate alternative mRNA splicing in the nucleus [33] but data from *Malat1* knockout mice show no developmental abnormalities and no clear changes in mRNA splicing [34]. As indicated by its name, MALAT1 has been associated with poor clinical outcomes in lung cancer [31]. However, gene inactivation studies in mice yielded conflicting results with studies showing both enhanced [35] and decreased [36] metastatic potential. Besides regulating miRNA, MALAT1 directs the polycomb repressor complex (PRC) to methylate and silence key genes [37]. MALAT1 also influences gene expression by binding and inactivating the Tead family of transcription factors [35]. Through its varied effects, MALAT1 regulates PI3K-AKT, MAPK, WNT and NF-κB signaling pathways [38], which influence critical biological processes like proliferation, inflammation, cell survival and metabolic function. While the study of Liang et al. [14] suggests that targeting MALAT1 could ameliorate allergic asthma, MALAT1 manipulation would need to be tightly controlled and directed specifically toward CD4^+^ cells. As indicated above, MALAT1 may exert protective effects depending on the cellular context so systemic inhibition could bring about deleterious off-target outcomes. The efforts of Liang et al. [14] advance the field of RNA biology by identifying a heretofore previously unknown role for MALAT1 in Th2-mediated inflammation. This underscores the importance of further studies to better delineate the biological effects of MALAT1 on inflammatory and metabolic responses implicated in tissue injury and repair.

## Abbreviations

BACE 1: β-site amyloid precursor protein cleaving enzyme 1
CTLA-4: cytotoxic T-lymphocyte antigen 4
IFN: interferon
MALAT1: metastasis-associated lung adenocarcinoma transcript 1
Th1/Th2: Thelper1/Thelper2
T-bet: T box protein expressed in T cells
